# Supplementary material for: Resolving the full spectrum of human genome variation using Linked-Reads
Source: Genome Res. 2019 Apr;29(4):635–45. doi: 10.1101/gr.234443.118 (PMC6442396; doi:10.1101/gr.234443.118)
Supplement: Supplemental Material [file supp_gr.234443.118_Supplemental_longranger-2.2.zip › longranger-2.2.tar.bz2!/lr2.2-public/lib/stan/cmdstan/stan/doc/api/html/prob_2traits_8hpp_source.html]

Stan: src/stan/prob/traits.hpp Source File


|  |  |
| --- | --- |
|  | Stan  2.5.0  probability, sampling & optimization |


- Main Page
- Related Pages
- Namespaces
- Classes
- Files

- File List
- File Members

All Classes Namespaces Files Functions Variables Typedefs Enumerations Enumerator Friends Macros Pages

- src
- stan
- prob

traits.hpp

Go to the documentation of this file.

1 #ifndef STAN\_\_PROB\_\_TRAITS\_HPP

2 #define STAN\_\_PROB\_\_TRAITS\_HPP

3

4 #include <stan/meta/traits.hpp>

5 #include <boost/math/tools/promotion.hpp>

6

7 namespace stan {

8

9  namespace prob {

10

29  template <bool propto,

30  typename T1=double, typename T2=double,

31  typename T3=double, typename T4=double,

32  typename T5=double, typename T6=double,

33  typename T7=double, typename T8=double,

34  typename T9=double, typename T10=double>

35  struct include\_summand {

36

42  enum {

43  value = ( !propto

44  || !stan::is\_constant<typename scalar\_type<T1>::type>::value

45  || !stan::is\_constant<typename scalar\_type<T2>::type>::value

46  || !stan::is\_constant<typename scalar\_type<T3>::type>::value

47  || !stan::is\_constant<typename scalar\_type<T4>::type>::value

48  || !stan::is\_constant<typename scalar\_type<T5>::type>::value

49  || !stan::is\_constant<typename scalar\_type<T6>::type>::value

50  || !stan::is\_constant<typename scalar\_type<T7>::type>::value

51  || !stan::is\_constant<typename scalar\_type<T8>::type>::value

52  || !stan::is\_constant<typename scalar\_type<T9>::type>::value

53  || !stan::is\_constant<typename scalar\_type<T10>::type>::value

54  )

55

56  };

57

58  };

59

60

61  }

62

63 }

64

65 #endif

stan::is\_constant

Metaprogramming struct to detect whether a given type is constant in the mathematical sense (not the ...

**Definition:** traits.hpp:43

traits.hpp

stan::prob::include\_summand

Template metaprogram to calculate whether a summand needs to be included in a proportional (log) prob...

**Definition:** traits.hpp:35

stan::prob::include\_summand::value

**Definition:** traits.hpp:43


---

[ Stan Home Page ]

*© 2011–2014,
Stan Development Team.*
